# SEEKING ANSWERS FOR UNCERTAINTIES: GATHERING AND ORGANIZING CARE HOME UNCERTAINTIES: THE SEARCH U STUDY

**DOI:** 10.1093/geroni/igad104.2858

**Published:** 2023-12-21

**Authors:** Reena Devi, Kirsty Haunch, Liz Graham, Karen Spilsbury, Anne Forster, Alys Griffiths, Carl Thompson

**Affiliations:** University of Leeds, Leeds, England, United Kingdom; University of Leeds, Leeds, England, United Kingdom; University of Leeds, Leeds, England, United Kingdom; University of Leeds, Leeds, England, United Kingdom; University of Leeds, Leeds, England, United Kingdom; University of Sheffield, Sheffield, England, United Kingdom; University of Leeds, Leeds, England, United Kingdom

## Abstract

**Introduction and method:**

Staff providing care to residents must manage uncertainties whilst using their judgement and making decisions about care. Residents and their relatives may also have questions and ideas about how care could be improved. It is important to capture uncertainties/questions and work on addressing these. We gathered uncertainties/questions from residents, relatives and staff before, and during COVID-19 through interviews, analysis of a COVID-19 WhatsApp discussion group for long-term-care (LTC), and a systematic literature review.

**Results:**

Questions and uncertainties gathered were categorised into 21 themes: 1. Direct care (personal care, sleep, care planning, medication management, nutrition) 2. Physical environment (safety, mobility, infection risk) 3. Mental health/well-being (relationships, socialising and meaningful activities, spirituality) 4. Resident individuality and dignity 5. End-of-life 6. Quality of work for care staff (reward and recognition, training and career opportunities, well-being, shift flexibility, workload) 7. Staff shortages 8. Promoting staff diversity 9. Care home interaction with wider healthcare services 10. Use of technology 11. Family engagement and communication with staff 12. COVID-19-related anxiety 13. Financial support with care home costs 14. Care homes and climate change and future sustainability 15. Valuing the contribution of care homes 16. Infection prevention control 17. Visiting care homes during COVID-19 18. COVID-19 safety for residents and staff 19. COVID-19 and the individual (symptoms and treatment) 20. Organisational impact of COVID-19 21. Information sharing and learning from COVID-19.

**Discussion:**

With LTC staff, residents and relatives the identified uncertainties/questions will be shortlisted; the prioritised list will direct future research.

